# Left atrial metastasis of renal cell carcinoma: a case report and review of the literature

**DOI:** 10.1186/1756-0500-7-520

**Published:** 2014-08-12

**Authors:** Kojiro Ohba, Yasuyoshi Miyata, Kensuke Mitsunari, Tomohiro Matsuo, Yasushi Mochizuki, Hideki Sakai

**Affiliations:** Department of Nephro-Urology, Nagasaki University Graduate School of Biomedical Sciences, 1-7-1 Sakamoto, Nagasaki, 852-8501 Japan

**Keywords:** Atrium, Metastasis, Renal cell carcinoma

## Abstract

**Background:**

Cardiac metastasis of renal cell carcinoma is an exceptional event, particularly when there is lack of inferior vena cava involvement. Indeed, only a few cases have been reported worldwide thus far. Moreover, discussion of treatment and follow-up strategies for cardiac metastasis of renal cell carcinoma is important because of the high risk of sudden death.

**Case presentation:**

We report the case of a 75-year-old Japanese man with metastatic tumor in the left atrium from renal cell carcinoma. He had a history of right renal cell carcinoma, for which he had undergone hand-assisted laparoscopic nephrectomy. Lung and bone metastases were detected after nephrectomy, and treatment with interferon-alpha was initiated. After disease progression, he was treated concurrently with targeted molecular therapy and radiotherapy for bone metastasis. After these therapies, a 42 × 24 mm mass was found on transthoracic echocardiography in left atrium without involvement of the right atrium or inferior vena cava. The provisional diagnosis was metastatic mass or myxoma, and surgical resection was performed. Histopathological examination led to a final diagnosis of metastatic tumor from clear cell renal cell carcinoma.

**Conclusion:**

Cardiac metastasis, metastasis to the left atrium in particular, is rare in patients with renal cell carcinoma. In our study, surgery of the cardiac mass was effective to avoid sudden death and quality of life decline resulting from heart failure. We describe this case and review cardiac metastasis of renal cell carcinoma.

## Background

Renal cell carcinoma (RCC) represents about 3% of all malignant tumors. Metastasis is a strong predictors in patients with RCC. Common sites of RCC metastasis are the lung, lymph nodes, bone, and liver. Conversely, cardiac metastasis of RCC is an exceptional event with only a few cases reported worldwide to date, although cardiac involvement via the inferior vena cava (IVC) thrombi is well-known. Moreover, discussion of treatment and follow-up strategies for cardiac metastasis of RCC is important because of the high risk of sudden death.

## Case presentation

A 75-year-old Japanese man was admitted to our hospital in June 2010 for consciousness disturbance. After admission, cerebral infarctions and a left atrium (LA) mass were identified on computed tomography (CT). He had previously been diagnosed with RCC and had undergone hand-assisted laparoscopic nephrectomy in October 2006 (pT2N0M0). Subsequently, multiple lung metastases and mediastinal lymph node metastases were detected on chest CT in April 2008. His Karnofsky performance status score was 100, and Memorial Sloan-Kettering Cancer Center risk classification was “favorable”. The patient was therefore treated with three doses per week of subcutaneous interferon-alpha at 5 million units. However, owing to progression of lung metastases and the appearance of a pubic bone metastasis, treatment was changed to sorafenib at 800 mg/day in August 2009. In January 2010, radiotherapy (total: 39 Gy in 13 fractions) was added owing to progression and pain of the pubic bone metastasis. At that time, sorafenib was changed to everolimus at 10 mg/day. In July 2010, a solid mass was found in the LA on routine CT of the lungs.

At the time of admission, vital signs were stable, with blood pressure at 132/84 mmHg and heart rate at 64 beats/min. Laboratory tests results indicated anemia (hemoglobin, 9.3 g/dL) and renal failure (creatinine, 2.0 mg/dL), and electrocardiography revealed sinus rhythm.

**Figure 1:**
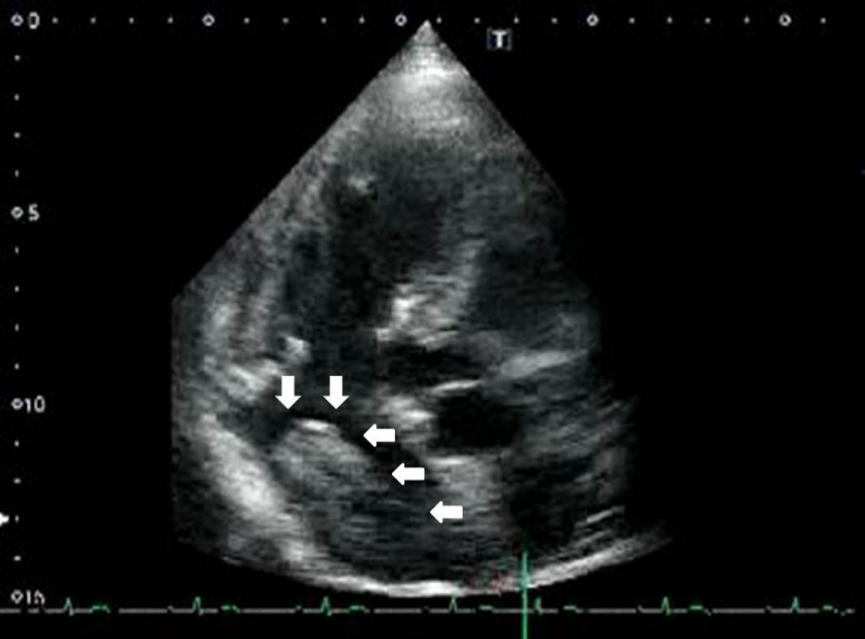
**Transthoracic echocardiography shows a 42 × 24 mm left atrium mass (indicated by arrowheads), moving without extension into the outflow tract.**

**Figure 2:**
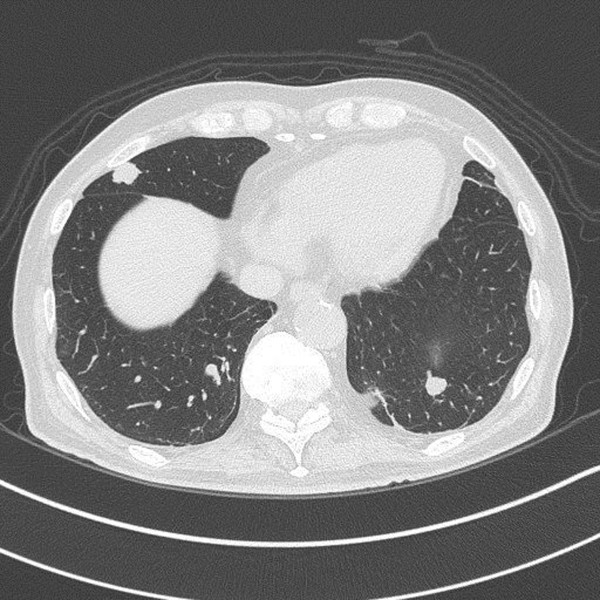
**Chest computed tomography reveals multiple lung metastases, but the left atrium mass is unclear.**

**Figure 3:**
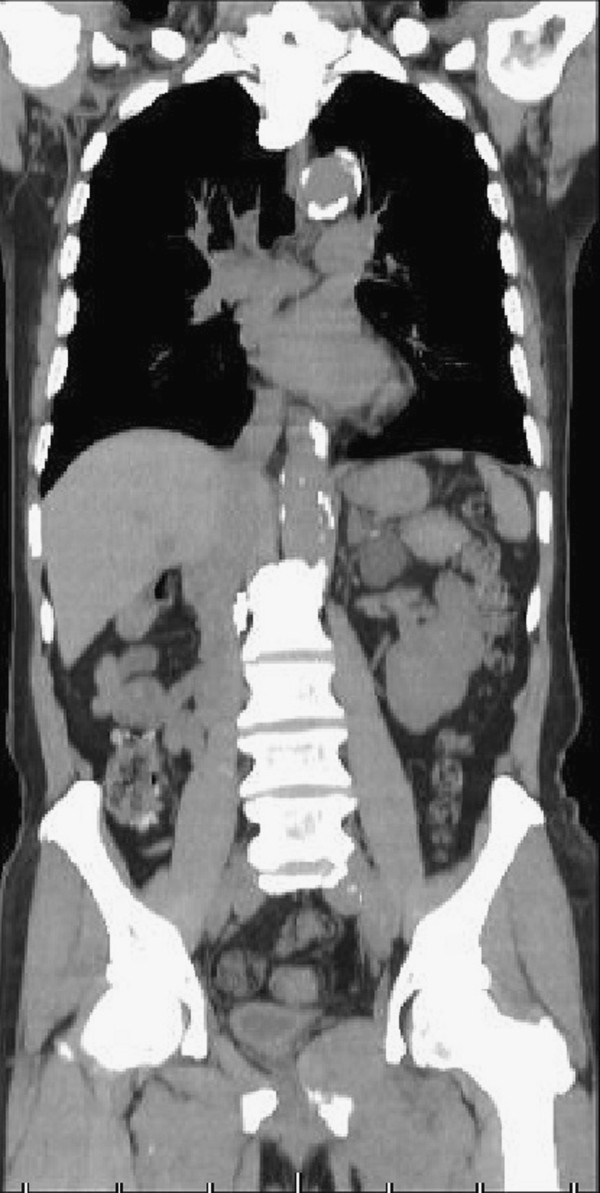
**There was no evidence of inferior vena cava involvement.**

Transthoracic echocardiography showed a 42 × 24 mm mass in the LA that moved without extension into the outflow tract (Figure 1). The right atrium and interatrial septum appeared normal. CT of the chest and abdomen revealed multiple metastatic tumors of the lungs, lymph nodes and pubic bone, but no IVC involvement, and the LA mass was unclear (Figures 2 and 3).

The appearance of the LA mass was consistent with myxoma, but the history of RCC raised the possibility of an intracardiac metastatic mass. Finally, the LA mass was surgically resected to prevent sudden death from acute heart failure or embolism resulting from tumor separation. Although the tumor had grown into the right superior pulmonary vein as the site of lymph node metastasis, the mass was completely resected.

Pathological assessment of the LA tumor revealed clear cell RCC that was diagnosed as a metastasis from the original RCC. After surgery, the patient did not experienced symptom of heart disease, such as dyspnea, chest pain, or syncope. However, multiple brain metastases were found subsequently, and the patient died from progressive disease 4 months postoperatively.

## Discussion

Cardiac metastases of RCC are rare. Actually, a study of 266 cardiac neoplasms found in 11,432 autopsy cases revealed just 3 cases originating of RCC [1]. Furthermore, cardiac metastases without IVC thrombi are not common. To the best of our knowledge, including present case, 31 reports have described cardiac metastasis of RCC without IVC involvement [2–12]. Therefore, we reviewed and analyzed these reports. LA metastasis appears to be particularly rare, and this represents just the third report of LA metastasis of RCC [7, 8]. In the 31 reports identified, metastasis to the heart alone and metastases to multiple sites together with cardiac involvement showed comparable frequencies (14 and 17 cases, respectively; Table 1) [2–12]. No clear difference in the frequency of laterality between the right and left heart was evident (16 and 14 cases, respectively with no bilateral cases).

**Table 1:** **Summary of previous reports of cardiac metastasis of renal cell carcinoma**

Multifocality	Single	14
	Multiple	17
Metastatic side	Right atrium	3
	Right ventricle	13
	Left atrium	3
	Left ventricle	10
	Left atrium and ventricle	1
	Bilateral atrium	1
Treatment	Surgery	14
	Cytokine	6
	Tyrosine Kinase inhibitor	2
	Unknown	9

These reports showed that right heart metastasis tended to be associated with absence of metastasis to other organs. Conversely, left heart metastasis tended to be associated with other organ metastases, although no significant correlation between disease side and multifocality was evident (p = 0.346) [2–12].

Cardiac metastases of RCC seem to occur through two mechanisms. The first is a venous hematogenous pathway through the renal vein to the right heart. In cases with isolated disease, delayed progression to the right heart, without involvement of the IVC, remains the most probable mode of metastasis through venous hematogenous microdissemination. The second mechanism involves a lymphatic pathway through the lymphatic vessels of the thorax, involving the carinal lymph nodes and parasternal lymph vessels. Reports have mentioned that drainage from the left heart wall passes through these lymph vessels, and lymphatic flow can be reversed by metastasis to the nodes [4, 13]. This hypothesis is supported by the fact that disseminated disease progression involves the supradiaphragmatic and mediastinal lymph nodes and the lungs, and the side of the heart involved more frequently is the left side.

The second mechanism of metastasis is more compatible with the present case, because the left side of the heart was affected by metastasis, the diseases was non-isolated, and lung metastasis was present.

Malignant cardiac tumors have been managed surgically in some patients, but no general consensus has been reached regarding treatment strategies for such disease. For carefully selected patients, resection of cardiac metastases has been used to provide symptom palliation, improved quality of life, and prolonged survival [14]. In recent years, Szmit et al. reported a case of heart failure caused by heart metastases of RCC successfully treated using receptor tyrosine kinase inhibitor [5]. In addition, Karakiewicz et al. reported the neoadjuvant setting using sunitinib to downstage an atrial thrombus [15]. Conversely, some reports have described cases of sudden death due to cardiac metastases [2, 4, 16].

In cases of isolated cardiac metastasis, surgical resection seems effective and offers favorable outcomes [6, 7]. In cases with metastases to multiple sites including the heart, improvement of heart failure and prevention of sudden death may be achieved using a combination of surgical treatment and targeted molecular therapy.

## Conclusion

We have reported a case of LA mass caused by metastatic RCC and have reviewed previous reports on the subject. Surgical excision of the cardiac mass is effective to avoid sudden death and decline in quality of life resulting from heart failure.

## Consent

Written informed consent was obtained from the patient’s son for publication of this case report and accompanying images. A copy of the written consent is available for review by the Editor of this journal.
